# Syndromic Surveillance in Bioterrorist Attacks

**DOI:** 10.3201/eid1109.050981

**Published:** 2005-09

**Authors:** Arnold F. Kaufmann, Nicki T. Pesik, Martin I. Meltzer

**Affiliations:** * Centers for Disease Control and Prevention, Atlanta, Georgia, USA

**Keywords:** syndromic surveillance, bioterrorist, anthrax, commentary

The article by Nordin et al. (*1*) in this issue of Emerging Infectious Diseases describes the use of syndromic surveillance to detect inhalational anthrax resulting from a hypothetical covert release of *Bacillus anthracis* spores at a major shopping mall. This study is an important evaluation of syndromic surveillance's utility in detecting an inhalational anthrax epidemic against a background of real patient presentations. Based on historical clinical data from a large health maintenance organization (HMO), the authors evaluated the sensitivity of a syndromic surveillance system to detect an incident by season of the year, day of the week when the release occurred, and attack rate in mall patrons.

Although numbers of persons exposed and becoming ill, as modeled in the study, are not specified, the effect can be inferred from the specified methods. On the basis of information from the mall's Web site (*2*) and the methods stated in the article, the number of cases associated with a 15% attack rate in mall visitors (115,000 daily average) and workers (12,000) would be ≈19,000 (if no additional exposures occurred after day of release). Of these patients, 59% would be from the metropolitan area in which the mall was located, an additional 6% would reside within a 150-mile radius of the metropolitan area, and the remainder would be from more distant points, including international visitors. Syndromic surveillance, with the HMO patient database, would detect 50% of such incidents by day 5, with only 20% detected by day 4. Lesser attack rates would notably lower the probability of detection. Even more problematic, the syndromic surveillance systems, as modeled, would fail to detect the outbreak in 13% of releases in summer and 47% of releases in winter. Performance would improve markedly with higher attack rates. After detection of an aberrant signal, the occurrence of a syndrome must be investigated to determine the cause, and exposure history of patients must be determined to discover the source. These investigations could result in additional delay before a targeted response could be mounted to prevent more illnesses. Such delays are problematic because the effectiveness of postexposure prophylaxis for inhalational anthrax is related to speed of implementation (*3*).

The authors point out that an astute clinician might diagnose inhalational anthrax in a patient before syndromic surveillance detected that an outbreak of some type was occurring. If, as the 15% attack rate scenario suggests, >100 patients had onset of illness on day 2 after exposure, a correct diagnosis could be established for at least 1 patient by day 4. By this time, hundreds of inhalational anthrax patients would be seen at hospitals, at least 1 day before the syndromic surveillance system, as modeled, would have a 50% probability of signaling the outbreak.

The issue now becomes whether or not syndromic surveillance can augment the public health response to an outbreak. For example, if a syndromic surveillance system allowed follow-up of individual cases, it might accelerate case finding and investigation into the source of infection. This potential role of syndromic surveillance was not included in the modeled scenario.

Syndromic surveillance systems, of the type modeled by Nordin et al., may be too slow to allow public health officials and policy makers to mount a sufficiently rapid postexposure prophylaxis campaign. Therefore, the ability of many current syndromic surveillance systems to rapidly detect bioterrorist attacks needs to be improved. Another reason to improve syndromic surveillance systems is that the systems may have public health value other than detecting bioterrorist attacks, such as tracking the course of seasonal diseases. We should not forget, however, that clinical care providers will continue to have a critical role in detecting bioterrorist attacks, and communications must be maintained with these first-line sentinels.
